# Increase in hippocampal water diffusion and volume during experimental pneumococcal meningitis is aggravated by bacteremia

**DOI:** 10.1186/1471-2334-14-240

**Published:** 2014-05-06

**Authors:** Jon G Holler, Christian T Brandt, Stephen L Leib, Ian J Rowland, Christian Østergaard

**Affiliations:** 1 Department of Infectious Diseases, Copenhagen University Hospital Hvidovre, Kettegaard Alle 30, Hvidovre, Copenhagen 2650, Denmark; 2 Department of Pulmonary and Infectious Diseases, Copenhagen University Hospital Hillerød, Dyrehavevej 29, Hillerød, Copenhagen 3400, Denmark; 3 Neuroinfection Laboratory, Institute for Infectious Diseases, University of Bern, Bern, Switzerland; 4 Biology Division, Spiez Laboratory, Federal Office for Civil Protection, Austrasse, Spiez CH-3700, Switzerland; 5 Department of Entomology, University of Wisconsin, Madison, USA; 6 Department of Clinical Microbiology, Copenhagen University Hospital Hvidovre, Kettegaard Alle 30, Hvidovre, Copenhagen 2650, Denmark

**Keywords:** Magnetic resonance imaging, ADC, Volume, Oedema, Meningitis, Apoptosis, Hippocampus

## Abstract

**Background:**

The hippocampus undergoes apoptosis in experimental pneumococcal meningitis leading to neurofunctional deficits in learning and memory function. The aim of the present study was 1) to investigate hippocampal apparent diffusion coefficient (ADC) and volume with MRI during the course of experimental pneumococcal meningitis, 2) to explore the influence of accompanying bacteremia on hippocampal water distribution and volume, 3) and to correlate these findings to the extent of apoptosis in the hippocampus.

**Methods:**

Experimental meningitis in rats was induced by intracisternal injection of live pneumococci. The study comprised of four experimental groups. **I**. Uninfected controls (n = 8); **II**. Meningitis (n = 11); **III**. Meningitis with early onset bacteremia by additional i.v. injection of live pneumococci (n = 10); **IV**. Meningitis with attenuated bacteremia by treatment with serotype-specific anti-pneumococcal antibodies (n = 14). T2 and diffusion weighted MR images were used to analyze changes in hippocampus volume and water diffusion (ADC). The results were correlated to ADC of the cortex, to ventricular volume, and to the extent of hippocampal apoptosis.

**Results:**

Both ADC and the volume of hippocampus were significantly increased in meningitis rats compared to uninfected controls (Kruskal-Wallis test, p = 0.0001, Dunns Post Test, p < 0.05), and were significantly increased in meningitis rats with an early onset bacteremia as compared to meningitis rats with attenuated bacteremia (p < 0.05). Hippocampal ADC and the volume and size of brain ventricles were positively correlated (Spearman Rank, p < 0.05), whereas no association was found between ADC or volume and the extent of apoptosis (p > 0.05).

**Conclusions:**

In experimental meningitis increase in volume and water diffusion of the hippocampus are significantly associated with accompanying bacteremia.

## Background

Bacteremia is an important complication in meningitis, and is present in 2/3 of all patients with pneumococcal meningitis [1]. We have previously studied the role of accompanying bacteremia in detail in experimental pneumococcal meningitis and have demonstrated that this complication resulted in an attenuated CSF pleocytosis [2], an interrupted cerebral autoregulation [3], and an increased mortality [4] as well as hippocampal apoptosis [5].

Also, an increase in brain ventricle size, blood brain barrier (BBB) leakage, and white matter apparent diffusion coefficient (ADC) was demonstrated in meningitis rats with increased bacteremia by using high resolution magnetic resonance imaging (MRI) [6]. MRI enables the *in vivo* study of pathophysiological alterations and complications during the course of meningitis and is a more sensitive method for the investigation of subtle changes as microinfarctions resulting from e.g. vasculitis than conventional CT imaging. MRI is also able to visualize blood–brain-barrier breakdown and identify regions of oedema due to fluid accumulation as well as cytotoxic oedema caused by cell swelling in ischaemia (for a review see [7]).

Apoptosis in the hippocampus is a characteristic histopathological finding in patients dying from bacterial meningitis [8]. Experimentally, a close correlation between the extent of hippocampal apoptosis and learning deficits has been thoroughly decribed [9,10]. In contrast to the use of high resolution MRI for studying pathophysiological changes in brain cortex and white matter [6,11], no information is available concerning MRI alterations in the hippocampus during the acute course of meningitis. To our knowledge, few studies have investigated hippocampal MRI changes in patients surviving meningitis [12,13]. In a study by de Jonge *et al*. [14], MRI alterations 8–14 years after the disease were sparse, and no correlation between learning deficits and hippocampal MRI-findings could be demonstrated.

The aim of the present study was to investigate morphology and changes in water diffusion in the hippocampus during the course of experimental pneumococcal meningitis *in vivo* using high resolution MRI. We also investigated the influence of accompanying bacteremia on the observed MRI based measurements and the extent of hippocampal apoptosis.

## Methods

### Experimental procedures

The present study is based on data obtained from two independent studies in experimental meningitis investigating the effects of bacteremia on the pathophysiology of pneumococcal meningitis [5,6]. All experimental protocols used in this study were approved by the Danish Animal Inspectorate (Dyreforsoegstilsynet). Rats were anaesthetized (midazolam (1.88 mg/kg, Dormicum®) and fentanyl/fluanisone (0.12 mg/kg, Hypnorm®)), and meningitis was induced by injecting adult male Wistar rats intracisternally with 3 × 10^4^ CFU *Streptococcus pneumoniae* serotype 3. The present study comprised of the following four experimental groups. **I**. Uninfected controls (n = 8); **II**. Meningitis (n = 11); **III**. Meningitis with early onset bacteremia (additional i.v. injection of 6 × 10^4^ CFU *Streptococcus pneumoniae* serotype 3 (n = 10)); **IV**. Meningitis with attenuated bacteremia (treated with serotype-specific anti-pneumococcal antibodies (n = 14)). Results on CSF and blood culture (CFU/ml) obtained 28 hours after infection has previously been published [6].

### MRI

MRI measurements were performed 28 hours after infection using a Varian SISCO 4.7 T imaging system and spectroscopy system. T1W, T2W, quantitative diffusion and dynamic MRI measurements were performed as previously described [11]. After imaging brains were harvested for histomorphometry.

### ADC in hippocampus

Quantitative diffusion measurements (along x, y, and z) were performed before the administration of contrast agent (echo time = 65 ms, repetition time = 1500 ms, matrix size = 128 × 128, field of view = 35 × 35 mm, number of transients = 1 (with b-values of 0, 185, 740, 1665 s/mm^2^; 16 contiguous slices). ADC maps were calculated from all 16 slices, as previously described in detail [6]. Measurements of regions of interest (ROI), were performed on 2 coronal slices covering either the total hippocampus area or only the dentate gyrus area using a histopathological specimen as template.

### Hippocampus volume

**Figure 1:**
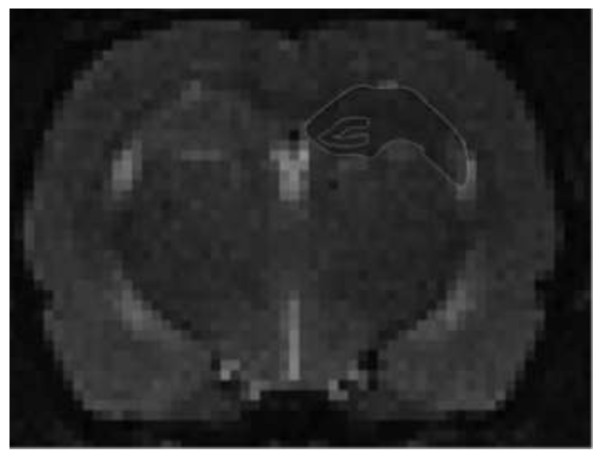
**T2W images showing placement of the ROI delineating the hippocampus and specifically the dentate gyrus in the rat brain.** The ROI was placed on two consecutive coronal brain slices. A mean ADC was measured using MIPAV.

Hippocampus volume was calculated using ROI covering the area of the hippocampus in 2 consecutive coronal slides. A mean ADC was calculated using MIPAV (http://mipav.cit.nih.gov/) (Figure 1). Drawing of ROI’s and calculation of ADC were performed by a person blinded to all other data.

Brain ventricle size, brain cortex ADC values, and BBB permeability (measured as the fraction of the cortex in which the contrast agent had delayed wash-out) have been published previously [6]. Data on hippocampus ADC, volume and hippocampus dentate gyrus ADC was generated for the present study.

### Hippocampal apoptosis

Hippocampal apoptosis data have, in part, been presented previously [5]. Apoptosis was assessed by a well established method as previously described [5,9,10,15]. In brief, fixed brains were examined for the occurrence of apotosis in the dentate gyrus of the hippocampus. Cryosections (45 μm thick) were Nissl-stained with cresyl violet. Cells exhibiting characteristic histomorphological features of apoptosis (condensed, fragmented dark nuclei, apoptotic bodies) were counted in 4 different slices spanning the hippocampus. Three visual fields in each of the two blades of the dentate gyrus were inspected. Each visual field was quantified using the following score: 0–5 cells = 0; 6–20 cells = 1; > 20 cells = 2. A mean value per animal was calculated. The apoptosis investigator was blinded to all data.

### Statistics

Kruskal-Wallis test with Dunn’s post test was used for comparison between the 4 study groups, whereas Mann Whitney test was used for comparison between 2 groups. Correlations within individual experimental groups were performed with Spearman rank test. p < 0.05 was considered significant.

## Results

### Microbiological results

CSF bacterial load (log_10_ CFU/ml) was comparable bewteen the incfected study groups (Meningitis rats median 6.4 [5 to 7.2]), rats with early onset bacteremia (median 6.2 [5.6 to 7.0]) and rats with attenuated bacteremia (median 5.3 [4.1 to 6.8]). The blood bacterial load (log_10_ CFU/ml) was significantly decreased among rats with attenuated bacteremia compared to the other meningitis study groups (median 0 [0 to 0.7] versus 2.3 [1.8 to 2.9] among meningits rats and 2.3 [2.0 to 2.7] in rats with early onset bacteremia (Mann–Whitney U-test p = 0.001 and p = 0.001, respectively)).

### MRI-findings in the hippocampus

A significant difference in ADC covering the total hippocampus area was demonstrated between the four experimental groups (Kruskal-Wallis test, p = 0.0001) with a significantly increased ADC in meningitis rats as compared to uninfected controls (Dunn’s post test, p < 0.05). ADC values were significantly lower in meningitis rats with attenuated bacteremia (Gr. IV) as compared to meningitis rats with early onset bacteremia (Gr. III, Dunn’s post test < 0.05). ADC in the ROI covering only the dentate gyrus was, however, not increased in meningitis rats as compared to uninfected rats (Kruskal-Wallis test, p = 0.19). Hippocampal ADC-values were significantly higher than ADC-values measured in the cortex in all three meningitis groups as well as in uninfected control rats (data not shown, Mann Whitney test, p < 0.001), (ADC and volume, see Table 1).

**Table 1:** ADC values of ROI covering the hippocampus and the dentate gyrus with corresponding volume changes in hippocampus

	**ADC hippocampus**	**ADC dentate gyrus**	**Volume (mm**^**3**^**) hippocampus**
Uninfected controls	71.6	71.4	12.1
	[70.0-73.0]	[67.5-74.2]	[11.0-12.9]
Meningitis	82.7	69.6	14.3
	[74.7-89.1]*	[65.2-80.7]	[13.2-15.8]*
Meningitis, early onset bacteremia	93.8	72.3	15.8
	[80.1-128.7]*,**	[66.1-82.1]	[13.7-18.1 ]*,**
Meningitis, attenuated bacteremia	75.7	70.8	11.8
	[71.8-82.2]	[67.5-74.5]	[10.4-14.1]

Data are presented as medians with quartiles.

Measurents of ROI, ADC and volume were performed on 2 coronal slices.

*p < 0.05 compared to the uninfected control group.

**p < 0.05 compared to the attenuated bacteremia group, Kruskal-Wallis test and Dunn’s post test.

The hippocampus volume differed significantly between the experimental groups (Kruskal-Wallis test, p = 0.0032) and was significantly higher in meningitis rats in comparison to uninfected rats (Dunn’s post test, p < 0.05). Meningitis rats with early onset bacteremia (Gr. III) had a significantly higher hippocampus volume when compared to meningitis rats with an attenuated bacteremia (Gr. IV, Dunn’s post test, p < 0.05).

**Figure 2:**
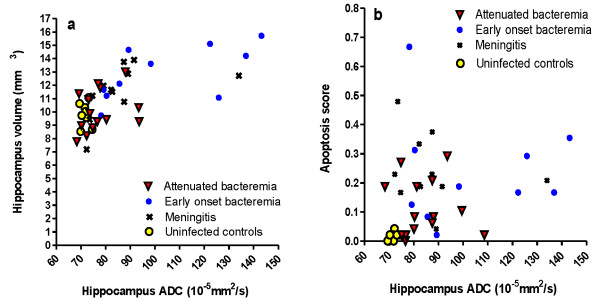
**Association between hippocampal ADC and hippocampal volume (a) and hippocampal apoptosis (b).** ADC of hippocampus was significantly correlated to volume (Figure 2a) in meningitis rats (Spearman Rank ρ = 0.75, p = 0.009) and in meningitis rats with early onset bacteremia (ρ = 0.65, p = 0.043), but not of statistical significance in meningitis rats with an attenuated bacteremia (ρ = 0.42, p = 0.14). No correlation between hippocampus ADC and apoptosis was observed (Figure 2b, p > 0.05).

There was a significant correlation between ADC in hippocampus and the corresponding volume measurement in meningitis rats (Gr. II, Spearman Rank ρ = 0.75, p = 0.009) and in meningitis rats with early onset bacteremia (Gr. III, ρ = 0.65, p = 0.043), but not of statistical significance in meningitis rats with an attenuated bacteremia (Gr. IV, ρ = 0.42, p = 0.14) (Figure 2a).

### Association between hippocampus MRI-findings, brain ventricle size and brain cortex ADC

ADC correlated significantly to the size of brain ventricles in meningitis rats with early onset bacteremia (Gr. III, ρ = 0.96, p < 0.0001) and in meningitis rats with attenuated bacteremia (Gr. IV, ρ =0.80, p = 0.0006), but not in meningitis rats (Gr. II, ρ = 0.21, p = 0.54). No significant correlation between hippocampus volume and brain ventricle size was observed among the individual meningitis groups (Gr. II, ρ = 0.11, p = 0.54, Gr. III, ρ = 0.57, p = 0.082, Gr. IV, ρ =0.24, p = 0.41). No significant correlation between ADC of hippocampus and cortex was observed (data not shown, p > 0.05).

### Association between MRI-findings and apoptosis in the hippocampus

No correlation between hippocampal ADC values (total or dentate gyrus ROI) and apoptosis scores could be demonstrated among the individual meningitis groups (p > 0.05) (Figure 2b). Similarly, no significant correlation between ventricle size and the degree of apoptosis was found among individual meningitis groups (p > 0.05).

## Discussion

Two types of brain oedema may develop and coexist during the course of meningitis, a vasogenic oedema resulting from increased BBB permeability and a cytotoxic oedema caused by cell swelling (i.e. hypoxia induced) [16].

The present results show that both ADC and volume in the hippocampus were significantly increased in meningitis rats compared to uninfected controls, and ADC and volume were positively correlated. An increase in ADC represents an altered water distribution in the extracellular compartment most likely representing a vasogenic oedema. We believe our findings to be in good agreement, since increased water diffusion and deposition of water in tissue would lead to tissue swelling, in this case documented by the significant volume increase of the hippocampus.

Theoretically, an increasing number of apoptotic neurons in hippocampus would allow for increased water diffusion and therefore an increased ADC [17], but despite the significant increases in both ADC and apoptosis in the hippocampus of rats with pneumococcal meningitis, we were unable to show a direct association. But whether this result is related to an insignificant fraction of apoptotic cells per visual field (less than one) or to a poor resolution of MRI when compared to histopatological examination still remains to be clarified. Since brain ventricles are located in close proximity to the hippocampus, and since an increase in ventricle size may reflect intracranial pressure changes, we hypothesized that the ventricle size may influence the pathophysiology of the hippocampus during pneumococcal meningitis. Our results show that there was a positive correlation between degree of vasogenic oedema (increased ADC) and brain ventricle size, but not between hippocampal volume and ventricle size. We believe that the lack of coherence between these results is due to the limited number of observations (type II error) in each group. Previously we have also shown that brain ventricle size was increased during the course of pneumococcal meningitis an was correlated with disease severity [11].

In contrast to the present findings in hippocampus, no significant increase in ADC values were shown previously in the cortex during experimental pneumococcal meningitis [6,11]. Also the results, showing that ADC values were significantly higher in hippocampus than in the cortex, suggest that the pathophysiology of pneumococcal meningitis may differ between brain regions. Indeed, the histopathological characteristics of brain damage from pneumococcal meningitis differ between hippocampus and other brain regions. Whereas apoptosis is observed in hippocampus, iscaemia, focal infarctions or abscess formations originating from vasculitis are the dominant feature in other brain regions [18]. Also, the processes leading to neuronal death is different between apoptosis and necrosis. Apoptosis is initiated by nucleolar scattering and cell shrinking contrary to focal ischaemia, where cell swelling is the initial step [15]. Previously MRI-studies on cerebral infarction have shown that in areas with focal iscaemia and infarctions, the development of necrosis is initiated by a decrease in water diffusion due to cell-swelling. In later stages, the shrinking necrotic neurons would allow for increased water diffusion and thereby a two-step transformation of ADC depending on the time for image acquisition [19].

Here we found that hippocampal ADC values and volume was higher in meningitis rats with increased bacteremia.

We have previously demonstrated an important role of accompanying bacteremia in experimental pneumococcal meningitis for both mortality [4] and brain pathophysiology (attenuated CSF pleocytosis [2], increased cerebral perfusion pressure and mean arterial pressure as well as an interrupted cerebral autoregulation [3], increased hippocampal apoptosis [5] and increased brain ventricle size, BBB leakage, and white matter apparent diffusion coefficient (ADC) [6]). As described above, there was a positive correlation between hippocampal ADC-values and size of brain ventricles. The present study was not designed to investigate a potential relationship between hippocampal ADC-values and cerebral and arterial pressure. However, alteration of the mean arterial pressure within the range ~75-125 mmHg either by controlled haemorrhage or norepinephrine therapy during experimental pneumococcal meningitis did not significantly alter ADC-values in the cortex (Michael Pedersen personal communication). Thus, the mechanism by which accompagnying bacteremia during meningitis may result in higher hippocampal ADC values is at present unknown.

## Conclusions

In conclusion, we have shown how increasing water diffusion, measured by ADC, in a well defined brain region is closely correlated with volume increase suggesting that ADC in bacterial meningitis efficiently reflects vasogenic oedema. The extent of apoptosis in the hippocampus could not be related to quantitative diffusion data obtained with MRI. In an experimental setting, where multiple pathophysiological changes affects gross morphology, histomorphometry remains the gold standard for the evaluation of neuronal injury in the hippocampus.

## Abbreviations

ADC: Apparent diffusion coefficient; CFU: Colony forming units; CSF: Cerebrospinal fluid; CT: Computed tomography; MRI: Magnetic ressonance imaging; ROI: Regions of interest.

## Competing interests

The authors declare that they have no competing interests.

## Authors’ contributions

JGH, CTB and CØ conceived the study, drafted the experimental protocol and manuscript. SLL carried out the histomorphometry of apoptosis in the hippocampus together with AB (*See Acknowledgments*). IJR contributed with technical support and analysis of MRI-findings, brain ventricle size and cortical ADC measurements. SLL and IJR contributed to writing, reviewing, and revising the paper. All authors interpreted the data and critically reviewed drafts of the manuscript. All authors edited and approved the final manuscript.

## Pre-publication history

The pre-publication history for this paper can be accessed here:

http://www.biomedcentral.com/1471-2334/14/240/prepub
